# Minimally invasive management of horseshoe kidney with polycystic kidney disease: A case report of successful tri-dimensional laparoscopic nephrectomy in a Puerto Rican female patient

**DOI:** 10.1016/j.ijscr.2025.111405

**Published:** 2025-05-03

**Authors:** Merary Z. Nazario-Perez, Amanda Torres-Arroyo, Rafael A. Brito-Sanchez, Gilberto Ruiz-Deya

**Affiliations:** a St. Luke's Hospital, Department of Graduated Medical Education, Research Fellow, 917 Av. Tito Castro, Ponce 00733, Puerto Rico; b St. Luke's Hospital, Urology Residency, 917 Av. Tito Castro, Ponce 00733, Puerto Rico; c Ponce Health Sciences University, School of Medicine Sala Ponce, 388 Calle Luis F, Ponce 00716, Puerto Rico; d Ponce Health Sciences University, Department of Surgery Sala Ponce, 388 Calle Luis F, Ponce 00716, Puerto Rico

**Keywords:** Polycystic kidney disease, Horseshoe kidney, End-stage renal disease, Laparoscopic nephrectomy, Three-dimensional laparoscopy, Case report

## Abstract

**Introduction:**

The coexistence of autosomal dominant polycystic kidney disease (ADPKD) with horseshoe kidney (HSK) is rare and presents significant surgical challenges due to fused anatomy and vascular variability. We report the first 3D laparoscopic bilateral nephrectomy case for ADPKD-HSK, demonstrating its advantages over conventional techniques.

**Case report:**

A 60-year-old woman with symptomatic ADPKD-HSK and end-stage renal disease presented with compressive symptoms. Comorbidities included diabetes, hypertension, hyperlipidemia, and AV graft dysfunction. A 3D laparoscopic approach enabled precise dissection, isthmus division, and safe removal of both kidneys. The patient experienced no intraoperative complications and recovered well postoperatively.

**Discussion:**

Nephrectomy in ADPKD-HSK is complex due to aberrant anatomy. While open surgery offers access, it carries higher morbidity. Laparoscopic nephrectomy reduces blood loss and shortens recovery—key benefits for transplant candidates. 3D laparoscopy improves visualization and vascular control at a lower cost compared to open and robotic techniques.

**Conclusion:**

3D laparoscopic bilateral nephrectomy is safe, effective, and cost-efficient for ADPKD-HSK. Further studies are needed to guide the surgical management of this rare condition.

## Highlights

• Horseshoe kidney (HSK) and autosomal dominant polycystic kidney disease (ADPKD) are rare congenital anomalies that pose significant surgical challenges.
• This is the first reported case of a 3D laparoscopic bilateral nephrectomy in a patient with ADPKD-HSK, incorporating bilateral kidney mobilization.
• The 3D laparoscopic approach enhanced surgical precision and depth perception, improving visualization of complex anatomical structures.
• This technique optimized anatomical space for future renal transplantation, minimizing operative risks and ensuring favorable postoperative outcomes.
• The findings highlight the potential of advanced 3D laparoscopy in managing complex renal pathologies, offering a minimally invasive alternative to traditional techniques.

## 1 Introduction

Horseshoe kidney (HSK) is a congenital anomaly characterized by the fusion of both kidneys at their lower poles, occurring in approximately 1 in 400 individuals [1]. Although often asymptomatic, HSK can lead to complications such as urinary obstruction, nephrolithiasis, infection, and end-stage renal disease (ESRD) [2]. The anatomical abnormalities of HSK, particularly the fused isthmus and atypical vasculature, pose significant surgical challenges.

Autosomal dominant polycystic kidney disease (ADPKD) is a genetic disorder caused by mutations in the PKD1 or PKD2 genes, leading to progressive cystic kidney enlargement and irreversible renal failure, frequently resulting in the need for transplantation [3]. The prevalence of PKD1 mutations is approximately 1 in 475 individuals, while PKD2 mutations occur in about 1 in 4000 [3]. Nephrectomy is often performed to alleviate symptoms or to create space for transplantation in patients with ADPKD [4].

The coexistence of ADPKD and HSK (ADPKD-HSK) is uncommon, and its surgical management is particularly complex due to distorted anatomy, extensive vascular involvement, and a fused isthmus [4]. Traditional surgical approaches, open nephrectomy (ON) and hand-assisted laparoscopic nephrectomy (HALN), present challenges in dissecting the horseshoe isthmus and mobilizing both kidneys [[5], [6], [7]]. A previously reported unilateral laparoscopic nephrectomy (LN) for ADPKD-HSK demonstrated the feasibility of a minimally invasive approach, but this case uniquely demonstrates the advantages of 3D laparoscopic bilateral nephrectomy [8].

Recent advancements in 3D laparoscopic technology have significantly improved surgical visualization and precision, making them an attractive option for complex renal surgeries [9]. The superior depth perception and enhanced imaging provided by 3D systems facilitate safer and more efficient dissection of intricate anatomical structures, reducing intraoperative risk and improving outcomes [9]. This report presents the first documented case of a 3D laparoscopic bilateral nephrectomy in a patient with ADPKD-HSK, emphasizing its advantages in complex surgical scenarios.

## 2 Case report

**Fig. 1:**
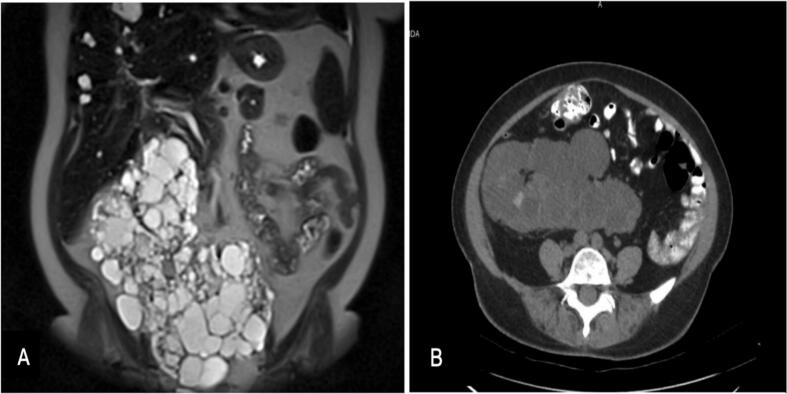
(A) MRI Abdomen with IV Contrast enhancement T2 weighted image (Coronal view) demonstrates a large, lobulated, multi-cystic structure in the midline, consistent with a horseshoe kidney exhibiting polycystic changes. The enlarged cystic kidneys seem to displace the surrounding structures, including the bowel and abdominal organs. (B) CT Scan of the Abdomen/Pelvis with PO and IV Contrast enhancement (Axial view) demonstrates hypodense cysts inside the horseshoe kidney.

A 60-year-old female presented with symptomatic ADPKD-HSK, with significantly enlarged kidneys causing compressive symptoms (Fig. 1) and kidney failure due to the natural progression of ADPKD. Laboratory findings consistent with ESRD and associated complications are summarized in Table 1, including elevated BUN and creatinine, anemia, electrolyte imbalances, and secondary hyperparathyroidism. After a multidisciplinary evaluation, laparoscopic nephrectomy was planned to create space for transplantation.

**Table 1:** Pre-operative laboratory workup.

Parameter	Result	Reference range
Renal function		
BUN	**62 mg/dL**	7–20 mg/dL
Creatinine	**12.25 mg/dL**	0.6–1.3 mg/dL
BUN/creatinine ratio	**5.1**	10–20
eGFR^a^	**3.1-3.8 mL/min**^a^	>60 mL/min

Electrolytes		
Sodium	142 mmol/L	135–145 mmol/L
Potassium	4.9 mmol/L	3.5–5.1 mmol/L
Chloride	100 mmol/L	96–106 mmol/L
Bicarbonate	26 mmol/L	22–29 mmol/L
Phosphorus	**5.3 mg/dL**	2.5–4.5 mg/dL
Corrected calcium	9.5 mg/dL	8.5–10.5 mg/dL
Calcium-phosphorus product	49	<55
PTH, intact (plasma)	**1516 pg/mL**	15–65 pg/mL

Hematology		
Hemoglobin	**10.5 g/dL**	12.0–16.0 g/dL
Red blood cell count	**2.92 mill/mcL**	4.20–5.40 mill/mcL
Hematocrit	**32.9 %**	36–46 %
MCV	**113 fL**	80–100 fL
RDW	13.9 %	11.5–14.5 %
White blood cell count	6.75 × 10^9^/L	4.0–11.0 × 10^9^/L
Platelet count	207 × 10^9^/L	150–400 × 10^9^/L
Iron	67 μg/dL	50–170 μg/dL
TIBC	330 μg/dL	240–450 μg/dL
Transferrin saturation	20 %	20–50 %

Dialysis adequacy		
Urea reduction ratio (URR)	79 %	65–80 %

a Glomerular filtration rate (GFR) was calculated using the MDRD equation and validated using the online calculator from MDCalc.com, with race not included in the estimation.

Her medical history included type 2 diabetes mellitus, hyperlipidemia, hypertension, left arteriovenous (AV) graft malfunction, and a herniated lumbar intervertebral disc. Past surgical procedures included partial hysterectomy, cesarean section, percutaneous angioplasty, and AV graft creation for dialysis. The patient was undergoing hemodialysis at the time of surgery. This case report has been reported following the SCARE 2023 criteria [10].

## 3 Operative procedure

The patient was positioned supine and secured to the operating table. After the induction of general anesthesia, pneumoperitoneum was established with a Veress needle. A 10-mm camera port and three working ports were introduced under direct visualization. A 3D laparoscopic camera provided enhanced visualization.

The right colon was mobilized medially to expose the retroperitoneum, followed by dissection of the upper pole of the right kidney. A similar mobilization was conducted on the left side. The horseshoe isthmus was identified and meticulously dissected, ensuring preservation of the inferior mesenteric artery. Segmental vessels were ligated utilizing laparoscopic staplers and advanced energy devices. Bilateral ureters were identified, ligated, and subsequently divided. The kidneys were placed within an endoscopic retrieval bag and extracted through an extended port site (Fig. 3). The resected specimen measured 22.6 × 15.3 × 11.4 cm and weighed 1260 g. No intraoperative complications were reported, and the estimated blood loss amounted to 200 cc. The procedure was conducted by a minimally invasive urologist at a high-volume center located in Ponce, Puerto Rico, with a total duration of 135 min.

## 4 Postoperative course

The patient's immediate postoperative recovery was uneventful. On postoperative day two, she developed AV graft thrombosis, which was managed with thrombectomy and temporary femoral vein catheter placement. Her postoperative course was also notable for a decline in hemoglobin from a preoperative level of 10.5 g/dL to 7.0 g/dL, requiring blood transfusion. This drop was attributed to the AV graft intervention and thrombectomy, as well as the surgical removal of the markedly enlarged polycystic kidneys. Using the ellipsoid formula (π/6 × length × width × thickness) and pathology-reported dimensions (22.6 × 15.3 × 11.4 cm), the estimated renal volume was approximately 2066.9 cm^3^ (equivalent to 2066.9 mL), supporting the degree of blood loss observed. A follow-up CT scan confirmed surgical success and anatomical suitability for future transplantation (Fig. 3). The patient was discharged in stable condition with planned outpatient follow-up.

## 5 Discussion

Autosomal dominant polycystic kidney disease on a horseshoe kidney (ADPKD-HSK) poses unique surgical challenges due to distorted anatomy, extensive vascular involvement, and technical complexities. Nephrectomy is often necessary for ADPKD to create space for transplantation [11]. In HSK, nephrectomy is usually reserved for malignancy, recurrent infections, or nephrolithiasis [12]. This case required a bilateral nephrectomy due to compressive symptoms and insufficient space for transplantation (Fig. 1). Given the rarity of ADPKD-HSK, no standardized surgical approach exists [13]. The fusion of the kidneys and aberrant vascularization complicates mobilization and isthmus dissection.

**Fig. 2:**
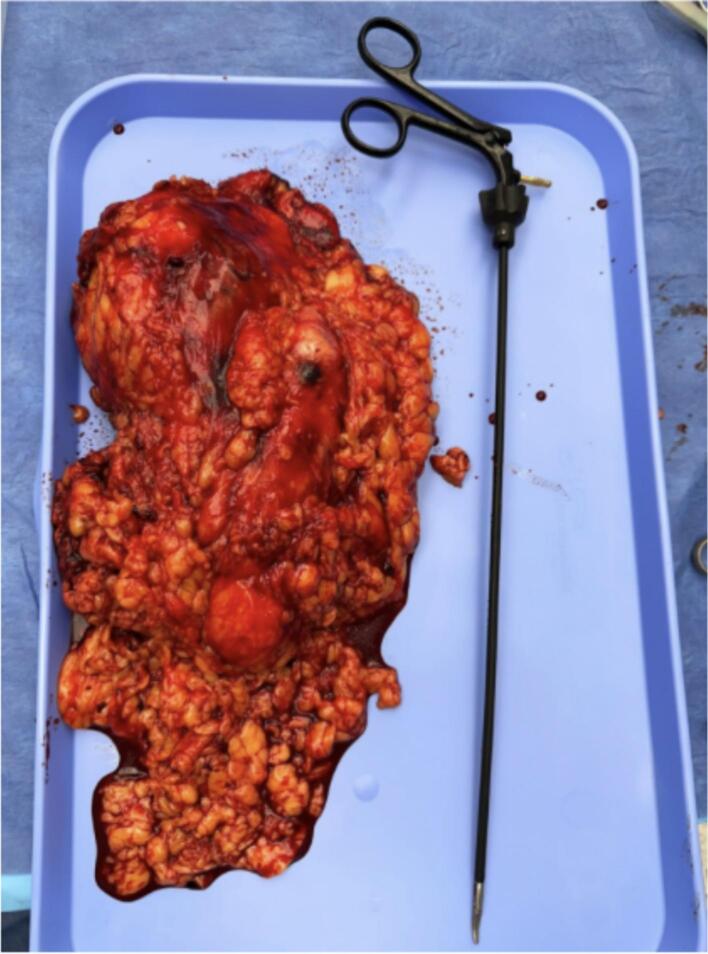
Photograph of the resected kidney.

Open nephrectomy (ON) provides superior visualization but is associated with greater morbidity and longer recovery [14]. In contrast, laparoscopic nephrectomy (LN) reduces blood loss, minimizes transfusion requirements, and shortens hospital stays [15]. A systematic review found that LN significantly lowers blood loss and transfusion rates, benefiting transplantation candidates by reducing the development of anti-human leukocyte antigen antibodies [16]. However, despite being less invasive, LN can be challenging for large kidneys due to limited working space and difficulties in organ manipulation. While ON allows for the faster removal of large kidneys, cyst decompression helps facilitate laparoscopic extraction [17]. In our case, despite the kidneys weighing 1260 g (Fig. 2), laparoscopic removal was successfully performed in 135 min, a duration comparable to reported times for laparoscopic nephrectomies involving kidneys larger than 10 cm. This suggests that 3D laparoscopic nephrectomy may offer comparable efficiency in select cases; however, further comparative studies are needed to evaluate operative time differences between standard laparoscopic and 3D laparoscopic approaches.

**Fig. 3:**
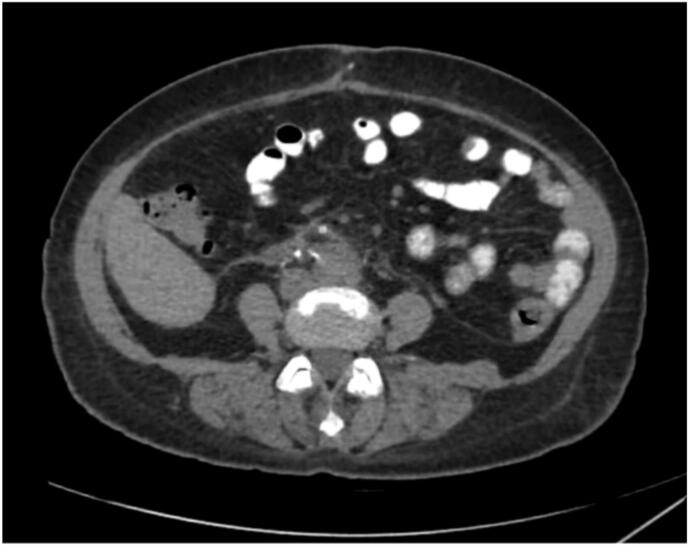
Postoperative CT scan of the abdomen and pelvis, enhanced with PO and IV contrast (axial view), demonstrates the successful removal of the horseshoe kidney and the cystic structures associated with polycystic kidney disease.

Hand-assisted laparoscopic nephrectomy (HALN) offers superior tactile feedback compared to conventional laparoscopic techniques; however, it is associated with larger incisions and increased blood loss relative to both standard laparoscopy and robotic-assisted laparoscopic nephrectomy (RALN) [18]. While RALN improves surgical dexterity and precision, it is often accompanied by longer operative times and increased costs [19]. The 3D laparoscopic system provides enhanced depth perception, magnified visualization, and improved contrast resolution [20]. Current evidence suggests that 3D laparoscopy significantly reduces the learning curve for acquiring laparoscopic skills, particularly for complex procedures. However, transitioning between 3D and 2D systems may present a secondary learning curve [21]. A summary of the advantages and disadvantages of these approaches is presented in Table 2.

**Table 2:** Comparison of Surgical Approaches for ADPKD-HSK Nephrectomy.

Surgical approach	Advantages	Disadvantages
Open Nephrectomy	‐ Direct visualization and control over aberrant vasculature. ‐ Ability to manage large ADPKD kidneys with extensive adhesions. ‐ Reliable hemostasis for isthmus division.	‐ Large incision with higher postoperative pain. ‐ Longer recovery time and hospital stay. ‐ Increased risk of surgical site infection. ‐ More blood loss compared to minimally invasive techniques.
Hand-Assisted Laparoscopic Nephrectomy (HALN)	‐ Better tactile feedback for dissecting large cystic kidneys. ‐ Shorter recovery than open surgery. ‐ Partial benefits of minimally invasive techniques.	‐ Compromised cosmesis (due to hand-port incision). ‐ Limited maneuverability in cases with multiple vascular anomalies. ‐ More blood loss than robotic or standard laparoscopy.
Robotic-Assisted Laparoscopic Nephrectomy (RALN)	‐ Superior instrument articulation for precise dissection of aberrant vessels and isthmus. ‐ Magnified 3D visualization. ‐ Less blood loss and faster recovery than open or HALN.	‐ Longer operative time. ‐ Higher cost and limited availability in some centers. ‐ Docking time can prolong the procedure.
Standard 2D Laparoscopic Nephrectomy	‐ Minimally invasive with small incisions. ‐ Less pain and shorter hospital stay compared to open or HALN. ‐ Lower blood loss than open surgery.	‐ Lack of depth perception. ‐ Difficult vascular dissection, especially in HSK anomalies. ‐ Steeper learning curve for complex renal anatomy.
3D Laparoscopic Nephrectomy (Proposed Approach)	‐ Enhanced depth perception, facilitating vascular dissection. ‐ Better anatomical visualization in patients with large cystic kidneys and complex vasculature. ‐ Lower cost compared to robotic-assisted surgery. ‐ Similar benefits to robotic surgery without additional docking time.	‐ Limited instrument articulation compared to robotic surgery. ‐ Operative time may be slightly longer than standard laparoscopy.

In this case, 3D laparoscopy facilitated precise isthmus dissection and vascular control, minimizing bleeding while optimizing kidney mobilization. Compared to ON, which allows easier organ mobilization but has higher morbidity, 3D laparoscopy combines enhanced visualization with minimally invasive benefits while avoiding the high costs of robotic surgery.

This is the first case report of a 3D laparoscopic bilateral nephrectomy in ADPKD-HSK with bilateral kidney mobilization. The approach optimized anatomical exposure and minimized risks, offering a reproducible strategy for similar cases. Unlike previous reports, incorporating 3D imaging enhanced vascular control and optimized the patient for future transplantation [8].

## 6 Conclusion

This case highlights the feasibility and potential advantages of 3D laparoscopic bilateral nephrectomy for ADPKD-HSK compared to other surgical alternatives. This procedure offers optimal visualization, enhanced depth perception, and precise vascular control while maintaining lower costs and faster recovery than traditional open techniques or robotic-assisted procedures. Given the rarity of ADPKD-HSK, future studies should evaluate the long-term outcomes of 3D laparoscopy in complex renal pathologies.

## Author contribution

Merary Z. Nazario-Perez: Contribution: Study concept, writing the paper, data collection, data analysis and interpretation.

Amanda Torres-Arroyo: Contribution: Writing the paper, data collection, data analysis.

Rafael A. Brito-Sanchez: Contribution: Writing the paper, data collection, data analysis.

Gilberto Ruiz-Deya: Contribution: Writing the paper, data collection.

## Consent

Written informed consent was obtained from the patient for publication and any accompanying images. A copy of the written consent is available for review by the Editor-in-Chief of this journal on request.

## Ethical approval

This research was reviewed and granted an exemption by the Institutional Review Board (IRB) of Ponce Health Sciences University (Protocol Number: 2501234229) under Federal regulations 45 CFR 46.101(b)(4). The exemption was granted as the study involves data recorded in such a manner that subjects cannot be identified, directly or indirectly, and the disclosure of this information would not place them at risk of criminal or civil liability, financial harm, employability consequences, or reputational damage.

## Guarantor

Merary Nazario-Perez.

Amanda Torres-Arroyo.

## Research registration number

This case report is not a “first in man” study.

## Funding

No funding was used for this case report.

## Conflict of interest statement

The authors declare no conflicts of interest in relation to this work.
